# The Role of the Skeletal Muscle Secretome in Mediating Endurance and Resistance Training Adaptations

**DOI:** 10.3389/fphys.2021.709807

**Published:** 2021-08-12

**Authors:** Aurel B. Leuchtmann, Volkan Adak, Sedat Dilbaz, Christoph Handschin

**Affiliations:** Biozentrum, University of Basel, Basel, Switzerland

**Keywords:** skeletal muscle, exercise, myokines, PGC-1alpha, endurance training, resistance training, secretome

## Abstract

Exercise, in the form of endurance or resistance training, leads to specific molecular and cellular adaptions not only in skeletal muscles, but also in many other organs such as the brain, liver, fat or bone. In addition to direct effects of exercise on these organs, the production and release of a plethora of different signaling molecules from skeletal muscle are a centerpiece of systemic plasticity. Most studies have so far focused on the regulation and function of such myokines in acute exercise bouts. In contrast, the secretome of long-term training adaptation remains less well understood, and the contribution of non-myokine factors, including metabolites, enzymes, microRNAs or mitochondrial DNA transported in extracellular vesicles or by other means, is underappreciated. In this review, we therefore provide an overview on the current knowledge of endurance and resistance exercise-induced factors of the skeletal muscle secretome that mediate muscular and systemic adaptations to long-term training. Targeting these factors and leveraging their functions could not only have broad implications for athletic performance, but also for the prevention and therapy in diseased and elderly populations.

## Introduction

Exercise, in its various forms, provides an array of physiological stimuli that evokes metabolic and molecular perturbations in skeletal muscle as well as many other organ systems. Training adaptations are structural and functional changes resulting from repeated exposure to these exercise-induced stimuli, leading to improved physiological capacity and decreased risk for morbidity and mortality (Egan and Zierath, 2013). The detailed molecular processes induced by different exercise stimuli are, however, still poorly characterized. In particular, it is incompletely understood how the signals induced by acute exercise bouts translate into specific adaptations following long-term training (Sanford et al., 2020). A sound understanding of the mechanisms underlying the adaptation to exercise training and the identification of molecular effectors of the adaptive response is essential—not only for individuals with athletic ambitions, but also to infer potential clinical implications and to develop novel therapeutic avenues for geriatric and diseased populations.

**FIGURE 1:**
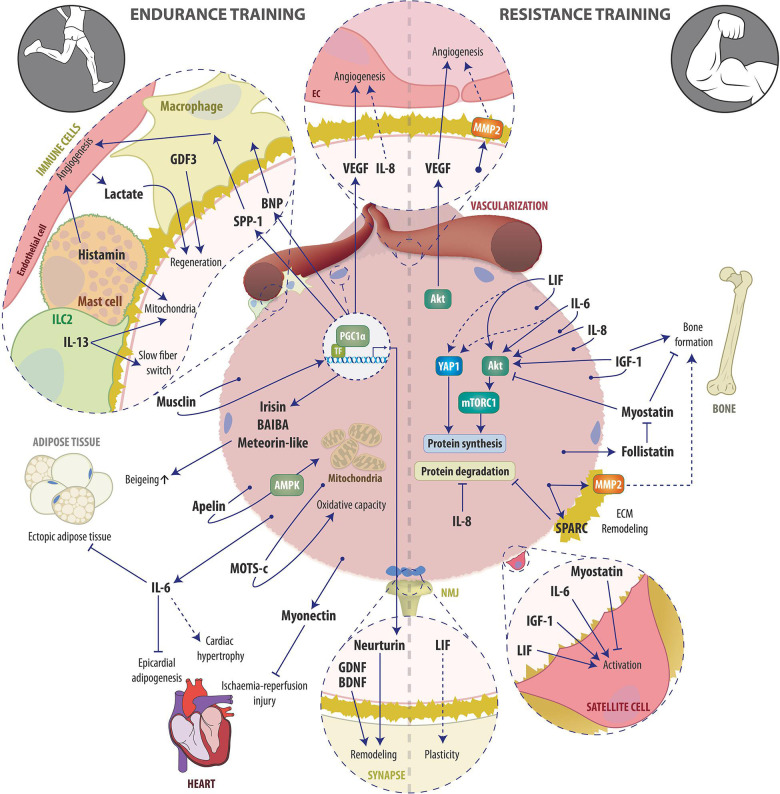
Schematic overview of examples of the endurance (left) and resistance (right) exercise-induced muscle secretome and its auto-, para-, and endocrine actions involved in training adaptation. See text for details. Abbreviations: AMPK, AMP-dependent protein kinase; Apelin, APJ Endogenous Ligand; BAIBA, β-aminoisobutyric acid; BDNF, Brain-derived neurotrophic factor; BNP, B-type or brain natriuretic peptide; EC, endothelial cell; ECM, Extracellular matrix; GDF3, macrophage-derived growth differentiation factor 3; GDNF, glial cell line-derived neurotrophic factor, IGF-1, Insulin-like growth factor-1; ILC2, innate lymphoid cells type 2; IL-6, Interleukin-6; IL-8, Interleukin-8; IL-13, Interleukin-13; LIF, Leukemia inhibitory factor; MOTS-c, mitochondrial ORF of the 12S ribosomal RNA type-c; MMP2, Matrix metalloproteinase-2; mTORC1, mammalian target of rapamycin complex 1; NMJ, Neuromuscular junction; PGC-1α, peroxisome proliferator-activated receptor γ coactivator 1α; SPARC, Secreted protein acidic and rich in cysteine; SPP-1, secreted phosphoprotein 1; TF, transcription factor; VEGF, Vascular endothelial growth factor; YAP1, Yes-associated protein 1.

Endurance and resistance exercise are the two extremes of a broad continuum of exercise modalities and elicit distinct but also overlapping training responses (Egan and Zierath, 2013). Acutely, both endurance and resistance exercise trigger the systemic release of a plethora of bioactive molecules such as proteins, metabolites and microRNAs (Whitham et al., 2018; Morville et al., 2020; Vechetti et al., 2021). The major physiological function of this release is thought to be the maintenance of metabolic homeostasis *i.e.*, the coordination of nutrient sensing, delivery, uptake and utilization, which requires an extensive crosstalk between almost every organ within the body (Murphy et al., 2020). Since the discovery of myokines, skeletal muscle has been appreciated as an endocrine organ, whose secreted factors are strongly involved in coordinating the metabolic network during acute exercise bouts in order to optimize energy substrate availability in working muscles and to sustain force production. Accordingly, during the last few decades, large efforts have been taken to elucidate the components of the muscle fiber secretome, with a particular focus on the regulation of the metabolic homeostasis during and after acute exercise bouts. In contrast, unraveling which of the acutely secreted factors are involved in mediating local and systemic adaptations to long-term training has received much less attention. In this review, we therefore summarize the current knowledge about secreted factors stimulated by endurance and/or resistance exercise that are contributing to long-term training adaptations (Figure 1).

## Endurance Training Adaptation

Endurance exercise is the training modality of choice when aiming for improvements in cardiorespiratory, cardiovascular and metabolic function (Mcgee and Hargreaves, 2020). Endurance exercise-induced stresses elicit multiple signaling events in skeletal muscle, heart and the respiratory system, which exert their effects on training adaptation in a cell-intrinsic manner. In addition, components of the skeletal muscle secretome, the entity of proteins, peptides, metabolites and other molecules (Weigert et al., 2014; Florin et al., 2020), released upon acute endurance exercise bouts, may also act as mediators of training adaptation through autocrine, paracrine and endocrine mechanisms. Many of the molecular adaptations in endurance exercise are controlled by the peroxisome proliferator-activated receptor γ coactivator 1α (PGC-1α). This coregulator protein is activated by exercise-linked signaling in muscle and in turn coordinates and orchestrates the transcription of a complex network encoding several biological programs linked to endurance training, including mitochondrial function, oxidative metabolism, calcium homeostasis and contractile properties of slow muscle fibers, or angiogenesis (Kupr and Handschin, 2015). Intriguingly, many myokines are also under the control of PGC-1α (Schnyder and Handschin, 2015). In the first part of this review, we will discuss how signaling factors are regulated and involved in the control of endurance training adaptation.

## Myokines

By definition, myokines are peptides that are produced and secreted by skeletal muscle fibers and subsequently exert auto-, para- or endocrine effects (Severinsen and Pedersen, 2020). A vast number of myokines have been described in various systems and paradigms, but only a subset of these is regulated by exercise (also called “exerkines”) (Laurens et al., 2020). In the following sections, the exerkines that are potentially involved in long-term endurance training adaptation are described.

### Interleukin-6

The discovery that skeletal muscle contributes to the exercise-induced increase in circulating interleukin-6 (IL-6) (Steensberg et al., 2000) initiated the concept of myokines and muscle as an endocrine organ. Despite initial skepticism about the muscle cell origin, there is now good evidence that IL-6 is transcribed, translated and released from myofibers during exercise (Hiscock et al., 2004; Whitham et al., 2012). The mechanism of secretion in contrast has not been entirely elucidated so far, although different candidate pathways have been proposed (Hojman et al., 2019). IL-6 serves primarily as a metabolic coordinator during acute exercise bouts by regulating lipolysis in adipose tissue (Van Hall et al., 2003) and hepatic glucose production (Febbraio et al., 2004) coordinated with substrate uptake and utilization in skeletal muscle (Chowdhury et al., 2020). The secretion of IL-6 is tightly linked to the metabolic state, e.g., pre-exercise muscle glycogen content or oral glucose availability (Starkie et al., 2001; Steensberg et al., 2001).

IL-6-driven lipolysis during acute exercise bouts may lead to improved body composition following endurance training over time. In fact, the reduction of visceral and epicardial fat tissue in response to 12 weeks of high-intensity endurance training was dependent on intact IL-6 signaling, at least in a cohort of obese and previously sedentary individuals (Christensen et al., 2019; Wedell-Neergaard et al., 2019). IL-6 is further known for its anti-inflammatory effects in both acute exercise bouts and exercise training. The acute effects are primarily due to the IL-6-triggered production of the cytokines IL-1 receptor antagonist and IL-10, which, amongst other exercise-induced factors, create an anti-inflammatory systemic environment (Pedersen, 2017). The long-term effects of training, in addition, may be positively influenced by the above-described reduction in visceral adipose tissue, a main contributor to the chronic systemic low-grade inflammatory state observed in the context of obesity (Severinsen and Pedersen, 2020). IL-6 may further be involved in endurance training-induced cardiac remodeling, as intact IL-6 signaling was required for training-linked left ventricle hypertrophy (Christensen et al., 2019). However, changes in stroke volume or end-diastolic volume (left ventricular ejection fraction) were not affected, and *V*O_2peak_, a marker for peak endurance capacity, was similarly ameliorated after endurance training in participants treated with the IL-6 receptor (IL-6R) antagonist tocilizumab or placebo (Wedell-Neergaard et al., 2019; Ellingsgaard et al., 2020). As a caveat: in the IL-6R blocking studies, it is not clear whether the long-term adaptations are in fact due to muscle-secreted IL-6 as other organs could contribute to the systemic increase during exercise. Furthermore, there is no evidence of sustained or enhanced IL-6 myokine production and release in endurance-trained muscle, implying that if long-term effects are mediated by muscle IL-6, they would be the consequences of repeated acute elevation and related signaling. In fact, the acute exercise-induced rise in systemic IL-6 levels and muscular IL-6 mRNA are diminished by training, although potentially counteracted by an enhanced muscular expression of the IL-6R in trained muscle (Severinsen and Pedersen, 2020).

### Interleukin-8

IL-8 is primarily known for its angiogenic activity and role as a chemoattractant in inflammatory processes (Harada et al., 1994; Keane et al., 1997). IL-8 acting as a potential myokine has been under investigation since *in vitro* studies have demonstrated that it is produced and secreted by muscle cells (De Rossi et al., 2000). Similar to IL-6, lower muscle glycogen content favors IL-8 transcription in human skeletal muscle, while changes in plasma levels due to exercise, conversely, seem to be glycogen-independent (Nieman et al., 2003; Chan et al., 2004). Moreover, a substantial increase in plasma IL-8 appears to require prolonged, potentially damaging muscle actions such as marathon- or ultramarathon-type of running (Nieman et al., 2002, 2003; Suzuki et al., 2003) and is less frequently observed or lower in magnitude following short term (Mucci et al., 2000) or less damaging muscular activity such as cycling or rowing (Henson et al., 2000; Chan et al., 2004). Considering the systemic stress and muscle damage induced by a marathon, the chemoattractant properties of IL-8 in inflammatory responses and immune cell activation are likely predominant in this context. Although muscle fiber-secreted IL-8 cannot be fully precluded to contribute to the chemoattraction of neutrophils and macrophages recruited for muscle repair, systemic IL-8 during strenuous exercise might also be derived from other cell types resident in skeletal muscle or beyond.

IL-8 signals *via* the CXC receptor 2 (CXCR2), which is expressed by endothelial cells (ECs) and affects EC proliferation and capillary tube organization (Li et al., 2003). Therefore, a potential role in training-induced capillarization has been proposed, which is supported by the observation that CXCR2 mRNA and protein levels are upregulated in vascular ECs upon exercise (Frydelund-Larsen et al., 2007), but conflicting with the impaired capillary outgrowth in human muscle explants exposed to higher levels of IL-8 (Amir Levy et al., 2015). The potentially pro-angiogenic effects of IL-8 on capillarization are likely to be dose-dependent and perhaps require the pulsatile nature of exercise training.

### Vascular Endothelial Growth Factor

Vascular endothelial growth factor (VEGF) is among the most important *pro*-angiogenic factors in many tissues. Accordingly, VEGF transcription and translation are highly induced in skeletal muscle upon endurance exercise (Jensen et al., 2004a; Olfert et al., 2016). Systemic levels of VEGF, on the other hand, often do not increase strongly during exercise, mainly because VEGF accumulates in the muscle interstitium acting on vascular ECs to trigger blood vessel formation (Höffner et al., 2003; Jensen et al., 2004a; Landers-Ramos et al., 2014). Because muscle cells can produce and secrete VEGF in response to electrostimulation (Jensen et al., 2004b), muscle fibers are thought to autonomously react to the stresses induced by exercise by secreting VEGF, which leads to enhanced capillarization following training and thereby facilitated energy substrate and oxygen supply as well as byproduct removal. Besides ample *post*-transcriptional regulation of VEGF stability, in many cell types VEGF induction is strongly regulated by the canonical hypoxia response pathway and hypoxia-inducible factor 1 alpha (HIF-1α)-controlled transcription (Arcondeguy et al., 2013). However, exercise-induced VEGF expression in skeletal muscle can be driven by additional, HIF-1α-independent mechanisms, orchestrated by PGC-1α interacting with the transcription factors estrogen-related receptor α (ERRα) and activator protein-1 (AP-1) (Arany et al., 2008; Baresic et al., 2014). In addition, PGC-1α-controlled angiogenesis may involve enhanced expression of platelet-derived growth factor (PDGF)-BB and angiopoietin 2 as well as secreted phosphoprotein 1 (SPP1). PDGF-BB recruits mural cells to support and encase the endothelium and angiopoietin 2 facilitates sprouting, while SPP1 instructs macrophages to signal to adjacent ECs, pericytes and smooth muscle cells thereby orchestrating angiogenesis in concert with VEGF (Arany et al., 2008; Rowe et al., 2014).

### Musclin

Skeletal muscle-derived musclin was first identified by screening cDNA libraries of mouse skeletal muscle for putative secreted proteins (Nishizawa et al., 2004). More than a decade later, Subbotina et al. (2015) established musclin as an exercise-controlled myokine whose production and systemic release is driven by Ca^2+^-dependent activation of Akt and subsequent nuclear exclusion of forkhead box O1 transcription factor (FoxO1), a known transcriptional inhibitor of the musclin-encoding gene osteocrin (*Ostn*) in skeletal muscle (Yasui et al., 2007). In sedentary mice, ubiquitous disruption of *Ostn* leads to impaired treadmill performance, reduced voluntary wheel running and lower succinate dehydrogenase activity in skeletal muscle, but had no effect on oxygen consumption during exercise or mitochondrial and respiratory complex protein content in skeletal muscle (Subbotina et al., 2015). In contrast, when exposed to short-term (5 days) treadmill training, all variables described above were significantly lower in *Ostn*-KO compared to wild-type (WT) animals, indicating impaired training adaptation in musclin deficient mice. Unfortunately, however, longer training interventions and pre- *vs. post*-training comparisons of running capacity and oxygen consumption were not performed in this study, which makes it difficult to draw firm conclusions about the importance of muscle derived musclin in endurance training adaptation.

The study by Subbotina et al. (2015) furthermore suggested that musclin acts in concert with atrial natriuretic peptide (ANP) to co-stimulate cGMP production in skeletal muscle, which has been linked to PGC-1α-dependent mitochondrial biogenesis (Nisoli et al., 2004). Exercise-induced increase in both cGMP and PGC-1α mRNA was blunted in muscles of *Ostn*-KOs (Subbotina et al., 2015). As endocrine musclin release increases systemic levels of various NPs by competing for their clearance receptor (Kita et al., 2009), exercise-induced musclin potentially modulates the action of these peptides. NPs have a wide spectrum of target tissues including skeletal muscle, heart, bone and kidneys. Thus, the endocrine release of musclin may, for example, increase cardiac NPs involved in heart remodeling, which in addition to the described effects on skeletal muscle could explain the reduced *V̇*O_2peak_ observed in endurance-trained *Ostn* KO compared to WT animals.

### B-Type or Brain Natriuretic Peptide

Brain natriuretic peptide (BNP) is mainly produced by cardiomyocytes in cardiac ventricles upon stretch to initiate signaling events that reduce blood pressure and blood volume (Goetze et al., 2020). As it is able to reduce fibrosis in the liver (Sonoyama et al., 2009) and potentially in the heart (Wang et al., 2004), coupled with the described immunomodulatory effect in macrophages (Chiurchiu et al., 2008), BNP is involved in inflammatory responses related to tissue repair and regeneration. In humans, plasma BNP increases in response to exercise (Ohba et al., 2001; Huang et al., 2002), but the relative contribution of different tissues to this increase is not exactly known. Because BNP expression is much higher in cardiac compared to skeletal muscle, the majority of circulating BNP likely originates from the heart. Nevertheless, by combining *in vitro* secretome analyses with *in vivo* experiments, BNP has been identified as a PGC-1α1/ERRα-driven myokine induced upon exhaustive endurance exercise in mouse skeletal muscle (Furrer et al., 2017). Together with other exercise and/or PGC-1α-regulated factors with paracrine immunomodulatory function in skeletal muscle, BNP may mediate the active engagement of skeletal muscle resident immune cells e.g., by orchestrating M1 to M2 transition in macrophages (Furrer et al., 2017). Whether and how the BNP-driven transient activation of macrophages after acute endurance bouts aids training adaptation in the long-term remains to be investigated. It seems, however, plausible that BNP-activated macrophages facilitate muscle repair and regeneration following acute exercise bouts and/or confer enhanced capacity to trained muscle to respond to exercise-induced insults by increasing their resident numbers. While the contribution of cardiac BNP most likely supersedes that of muscle BNP systemically, the latter could also have autocrine functions in skeletal muscle, based on the widely described actions of NPs on skeletal muscle (Miyashita et al., 2009; Engeli et al., 2012; Subbotina et al., 2015).

### Apelin

Apelin (short for APJ Endogenous Ligand) was first described as a peptide purified from bovine stomach extracts that binds to the orphan APJ G protein-coupled receptor (GPCR), now known as apelin receptor (Tatemoto et al., 1998). Both the apelin peptide and its receptor are expressed in many tissues including different brain regions and insulin-responsive tissues such as adipose tissue, skeletal muscle, heart and liver (Castan-Laurell et al., 2012). Apelin has long been known for its adipokine function, induced by insulin and modulating glucose and lipid metabolism as well as insulin sensitivity, which is why pharmacological targeting of the apelin receptor gained much interest for the treatment of type two diabetes (T2D) and metabolic diseases (Castan-Laurell et al., 2012). However, skeletal muscle-derived apelin and its responsiveness to exercise has only recently started to be explored, after a link between endurance training, muscular apelin expression and increased plasma apelin levels has been proposed (Besse-Patin et al., 2014; Fujie et al., 2014). In a comprehensive series of experiments, Vinel et al. (2018) investigated the muscular contribution to exercise-induced plasma apelin as well as the resulting local and systemic effects. *In vitro*, apelin was found to be secreted by contracting human myotubes, and in mice, hindlimb venous-arterial difference of apelin increased following muscle contractions induced by sciatic nerve stimulation. Moreover, skeletal muscle-specific knockdown of apelin expression through viral vectors did not affect resting levels, but impaired exercise-induced elevation of plasma apelin, reaffirming the strong contribution of skeletal muscle to plasma apelin during exercise. The muscle apelin knockdown also led to atrophy and reduced grip strength, grid-hanging time and treadmill performance, all of which were rescued with daily apelin treatment. While apelin supplementation had synergistic effects on endurance training and further improved fatigue resistance, muscle-specific overexpression phenocopied the functional effects of exercise training concomitant with an increased mass of overexpressing muscles. Regarding potential underlying mechanisms, Vinel et al. (2018) proposed an autocrine regulatory loop, in which local apelin production enhances muscular endurance by increasing intramyofibrillar mitochondrial number and function in part *via* AMP-dependent protein kinase (AMPK) activation. A previous study in insulin-resistant high-fat diet mice had already suggested a role for the apelin-AMPK axis in skeletal muscle mitochondrial biogenesis and oxidative capacity, as both were augmented by apelin treatment in an AMPK-dependent manner (Attané et al., 2012).

Apelin/apelin receptor signaling plays a key role in the development and organization of vascular networks, as evidenced in various *in vitro* and *in vivo* models (Masri et al., 2004; Kasai et al., 2008; Kidoya et al., 2008, 2015; Kunduzova et al., 2008). In addition, compared to WT animals, apelin-transgenic mice exhibit increased EC numbers in skeletal muscle when fed a high-fat diet, while animals on a standard diet displayed higher oxygen consumption and upregulated mRNA expression of endothelium-specific receptor tyrosine kinase 1 and 2, which are important for vascular maturation (Yamamoto et al., 2011). Mechanistically, apelin signaling pushes ECs into a *pro*-angiogenic state by enhancing intracellular glycolytic activity, a process mainly driven by PFKFB3 and c-MYC, two central regulators of EC metabolism (De Bock et al., 2013; Wilhelm et al., 2016; Helker et al., 2020). The apelin protein required for the *pro*-angiogenic switch, however, may be primarily derived from tip ECs for autocrine stimulation, as hypoxia can directly trigger apelin transcription in ECs by activating HIF-1α, which subsequently binds to hypoxia-responsive elements present in the apelin gene (Cox et al., 2006; Eyries et al., 2008; Del Toro et al., 2010). Similarly, during skeletal muscle regeneration, apelin is secreted together with oncostatin M and periostin by ECs and myogenic progenitor cells to couple myo- and angiogenesis (Latroche et al., 2017). However, no study has so far investigated whether muscle fiber secreted apelin could mechanistically contribute to the formation of capillaries in exercise-trained muscle.

### Myonectin

During the characterization of the metabolic function of complement component 1q/TNF-related protein (CTRP) family members, CTRP15 (now referred to as myonectin) was identified as a potential myokine (Seldin et al., 2012). In mice given access to running wheels for 2 weeks or subjected to 4 weeks of treadmill training, circulating myonectin levels as well as skeletal muscle gene and protein expression increased substantially (Seldin et al., 2012; Otaka et al., 2018). Furthermore, transcriptional activation of myonectin is induced by raising intracellular cAMP or Ca^2+^ levels through either muscular activity or pharmacologic agents (Seldin et al., 2012). Since myonectin is poorly expressed in tissues other than skeletal muscle, the main source of the training-induced increase in circulating levels is considered skeletal muscle (Seldin et al., 2012; Otaka et al., 2018). In a combination of *in vitro* experiments and studies in mice treated with recombinant protein, systemic myonectin was shown to act on the liver to increase the uptake of free fatty acids by upregulating local lipid binding and transport proteins (Seldin et al., 2012) and to suppress autophagy by activating the mammalian target of rapamycin complex 1 (mTORC1) pathway (Seldin et al., 2013).

Exercise mitigates ischaemia-reperfusion (IR) injury, which occurs after acute ischemic events such as myocardial infarction or suppression of blood flow during cardiac surgery (Moreira et al., 2020). Besides intracellular stress-defense mechanisms triggered directly in cardiomyocytes, the protective effect of exercise on IR injury may additionally be mediated by the exercise-induced myonectin release from skeletal muscle. Mice deficient for the myonectin gene displayed larger myocardial infarct sizes, cardiac dysfunction and more apoptotic cardiomyocytes compared to WT animals, while transgenic overexpression further reduced myocardial damage after IR (Otaka et al., 2018). Intriguingly, endurance training decreased infarct size after IR in WT, but not in myonectin KO mice, supporting the notion that the training-induced elevation of circulating myonectin confers this protective effect (Otaka et al., 2018).

Regarding human myonectin, there are so far only reports about plasma levels, and evidence as to the effect of both acute and chronic exercise is conflicting. In heterogeneous populations, some studies observed an increase while others reported either no change or even a decrease in circulating myonectin (Lim et al., 2012; Pourranjbar et al., 2018; Kamiński et al., 2019; Bahremand et al., 2020). Thus, more research is required, including studies on muscle biopsies, to help elucidating the role of human myonectin in general. Moreover, even though loss of myonectin has no effect on running performance in untrained mice (Little et al., 2019), there are no data available on *post*-training performance to conclude about potential impairments in training adaptation.

### Brain-Derived Neurotrophic Factor and Neurturin

Brain-derived neurotrophic factor (BDNF) signals *via* tropomyosin receptor kinase B (TrkB) and p75^*NRT*^ receptors and is a member of the neurotrophin (NT) family, a class of secretory factors that regulate survival, growth and function of neuronal cells. Accordingly, BDNF and its receptors are highly expressed in various brain regions (Huang and Reichardt, 2001). BDNF has been consistently reported to increase in the circulation in response to exercise [e.g., (Ferris et al., 2007; Saucedo Marquez et al., 2015)], with the brain or blood platelet precursors (megakaryocytes) likely to constitute the largest contributors to this increase (Rasmussen et al., 2009; Chacón-Fernández et al., 2016). However, both BDNF transcription and translation are induced in skeletal muscles during exercise, and the BDNF protein appears to be secreted, although only in relatively small amounts (Pedersen et al., 2009; Ogborn and Gardiner, 2010). Therefore, muscle-derived BDNF is thought to be mostly involved in autocrine and paracrine signaling e.g., to promote muscle fiber fat oxidation *via* AMPK activation (Matthews et al., 2009) and to regulate muscle development and regeneration, at least when SC-derived (Mousavi and Jasmin, 2006; Clow and Jasmin, 2010; Miura et al., 2012). In addition, a potential trophic action of muscle-derived BDNF on innervating motor neurons (MNs) *via* retrograde signaling has been proposed (Koliatsos et al., 1993). Surprisingly, even though BDNF induction seems to be a physiological response to endurance exercise, muscle-specific deletion of the *Bdnf* gene increases running performance, at least in part due to a neuromuscular junction (NMJ) remodeling associated with a myosin heavy chain (MyHC)-IIB to MyHC-IIX muscle fiber transition (Delezie et al., 2019). However, the role of BDNF induction (or repression) in training-induced remodeling of muscle fibers or their innervating MNs through auto- and paracrine actions, respectively, remains to be explored.

Many chronic diseases including T2D and cardiovascular disease, but also neurological disorders such as impaired cognition, dementia and depression are associated with lower circulating BDNF levels, and all these diseases can be mitigated with exercise interventions (Pedersen et al., 2009). Moreover, in mouse models, exercise-induced cathepsin B release from skeletal muscle elevate the abundance of BDNF in the hippocampus, which was linked to neuroprotection and improved memory function (Moon et al., 2016). The cathepsin B-mediated muscle-brain crosstalk is further supported by human data, as training induced cathepsin B transcription and systemic protein levels correlated with hippocampus-dependent memory function in healthy young adults (Moon et al., 2016). For more detailed information on muscle-brain crosstalk in the context of exercise, the interested reader is referred to a recent review on the topic (Delezie and Handschin, 2018).

Neurturin, a member of the glial cell line-derived neurotrophic factor (GDNF) family, is a PGC-1α1-regulated myokine and involved in neuromuscular synapse maturation during development and regeneration (Baudet et al., 2008). Accordingly, neurturin promotes neuromuscular junction (NMJ) formation in an *in vitro* microfluidics cell co-culture system (Mills et al., 2018b). Moreover, recent evidence indicates that neurturin could be one of the PGC-1α1 effector myokines that couples increased muscle oxidative capacity to corresponding changes in MN properties (Correia et al., 2021). While increased muscular expression after endurance exercise was detected in mice and humans (Schlittler et al., 2019; Correia et al., 2021), ectopic expression of neurturin in mouse skeletal muscle was sufficient to induce a shift toward slower MN and NMJ features, while at the same time, the muscles of these mice displayed a phenotype similar to PGC-1α1 overexpressing animals. Like BDNF, muscle-derived neurturin might primarily act in an auto- and paracrine manner, as it was undetectable in the plasma of overexpressing mice (Correia et al., 2021).

On the muscle side, neuromuscular adaptations to exercise training likely reflect the integration of the local relative abundance of various neurotrophic factors and their receptors. For example, GDNF is induced by exercise in rat skeletal muscle and has been implicated in enhanced NMJ endplate area in various training paradigms (Nguyen et al., 1998; Gyorkos and Spitsbergen, 2014; Gyorkos et al., 2014). Besides BDNF, other neurotrophic factors (NTs) such as nerve growth factor (Capsoni et al., 2000), NT-3 (Ernfors et al., 1994; Klein et al., 1994) or N-4/5 (Belluardo et al., 2001) are found in skeletal muscle and exert important functions in neuromuscular physiology. However, their regulation by exercise as well as their potential functions in the training response in rodents or humans remain largely elusive (Lippi et al., 2020). Collectively, however, the regulation, secretion and function of such factors imply a surprisingly broad retrograde signaling from skeletal muscle that leads to a remodeling of the NMJ and potentially MN phenotype, complementing the top-down control of the muscle phenotype by MN signaling.

## Adipose Tissue Beigeing Factors

In rodents, endurance exercise stimulates the muscular secretion of the PGC-1α-controlled factors β-aminoisobutyric acid (BAIBA) (Roberts et al., 2014), irisin (Boström et al., 2012) and meteorin-like (Rao et al., 2014), which all have the ability to promote brown fat activity and/or induce white adipose tissue beigeing, thereby significantly affecting energy expenditure. At a first glance, it appears counterintuitive that exercise, which increases energy demands and boosts heat dissipation, would induce signaling events that lead to mitochondrial uncoupling and thus reduced substrate utilization efficiency as well as heat production. Based on the observation that shivering-induced irisin secretion is similar in magnitude to the exercise-stimulated secretion, it has been hypothesized that exercise-induced adipose tissue browning may have evolved from shivering to aid cold exposure-induced thermogenesis (Lee et al., 2014). Irisin has also been linked to remodeling of bone tissue, cardiac protection, synaptic plasticity and memory, as well as neurogenesis, neuroinflammation and neurodegeneration in the brain (Ma and Chen, 2021). However, questions regarding physiological circulating levels in humans pre- and *post*-exercise remain to be addressed (Flori et al., 2021).

Similarly, conflicting reports about plasma levels of human BAIBA in response to exercise describe either an increase following an acute bout (Stautemas et al., 2019), while others failed in this attempt (Morales et al., 2017). The inconsistency in findings may have several reasons, including a gene polymorphism of an aminotransferase affecting baseline levels of total BAIBA or the fact that BAIBA has a chiral center and therefore two different enantiomers, which may lead to interindividual differences in their relative distribution and hence the relative change upon exercise (Stautemas et al., 2019).

Human meteorin-like plasma levels were significantly increased after exercise in temperate (24–25°C) and warm (36.5–37.5°C) water, but decreased in cold (16.5–17.5°C) water in a cohort of overweight women (Saghebjoo et al., 2018). In mice, a recent study reported that meteorin-like facilitates skeletal muscle repair by activating signal transducer and activator of transcription 3 (STAT3) signaling in macrophages to induce insulin-like growth factor 1 (IGF-1) production, which in turn had a direct effect on muscle SC proliferation (Baht et al., 2020). However, in this context, meteorin-like was released from infiltrating macrophages rather than resident cells or muscle fibers.

## Mitochondrial-Derived Peptides

Mitochondria possess a distinct circular genome that gives rise to 13 oxidative phosphorylation complex subunits. However, short open reading frames (sORF) have recently been discovered to encode additional biologically active small peptides (<100 amino acids), collectively referred to as mitochondrial-derived peptides (MDPs) (Kim et al., 2017). One of these MDPs, mitochondrial ORF of the 12S ribosomal RNA type-c (MOTS-c), activates similar signaling pathways as exercise and, when administered exogenously, promotes exercise-like adaptations (Lee et al., 2015; Kim et al., 2018). As a new class of circulating signaling molecules, skeletal muscle-produced and -secreted MDPs could potentially be involved in training adaptation. Indeed, both mice and human participants showed increased MOTS-c expression in skeletal muscle as well as increased circulating levels upon acute endurance exercise (Reynolds et al., 2021; Yang et al., 2021). Similarly, the MDP humanin increased in muscle and plasma of healthy young men following acute high-intensity cycling. This response was preserved, yet tended to be lower following short-term training (Woodhead et al., 2020). In the same study, electrical stimulation of isolated mouse muscle rapidly increased intramuscular humanin levels, supporting the notion of skeletal muscle being a source of circulating MDPs during exercise. However, the intracellular mechanisms regulating MDP production and secretion during exercise are still unknown, even though a responsiveness to signals associated with cellular energy stress occurring in exercised muscles is conceivable (Merry et al., 2020). Moreover, despite the training-like effects of systemic MOTS-c treatment on endurance performance in mice (Reynolds et al., 2021), the role and molecular targets of exercise-induced MDPs as well as their auto-, para- and endocrine relevance in mediating physiological training adaptation has yet to be established. Kumagai et al. (2021) suggested that MOTS-c could act as a myostatin inhibitor by increasing Akt activity *via* activation of mTORC2 and inhibition of phosphatase and tensin homolog (PTEN) through casein kinase 2 (CK2) activation. However, whether systemically administered MOTS-c modulated mTORC2 and CK2 activity in skeletal muscle by binding to an extracellular receptor, through direct interaction or by other means could not be determined. While putative cell-surface receptors for humanin have been suggested (Lee et al., 2013), extracellular receptors for MOTS-c or other MDPs are still elusive. Although more research has to be conducted, thus far it seems more likely that intracellular interactions dominate the effects of MDPs on metabolic homeostasis in the context of exercise training (Kim et al., 2018).

## Succinate

TCA cycle intermediates such as fumarate, malate, citrate and succinate have long been known to accumulate in skeletal muscle during exercise (Sahlin et al., 1990; Gibala et al., 1997). Of these, succinate is further released into the blood stream in substantial amounts (Hochachka and Dressendorfer, 1976). On target cells, succinate acts as an extracellular signaling molecule by binding to the GPCR succinate receptor 1 (SUCNR1) (He et al., 2004; Regard et al., 2008) and, in muscle fibers, stimulates monocarboxylate transporter (MCT)1-facilitated, pH-gated release of succinate (Reddy et al., 2020). A wide array of physiological and pathological processes have now been described to be, at least in part, regulated by extracellular succinate and/or SUCNR1 signaling including energy expenditure, inflammation, blood pressure and ischemia (Tretter et al., 2016; Mills et al., 2018a).

To evaluate the role of succinate signaling in training-induced muscle remodeling, Reddy et al. (2020) subjected SUNCR1 KO mice to a progressive resistance wheel-running regimen. After 3 weeks, SUNCR1 KOs had accumulated larger running distances compared to WT animals, indicating a superior endurance training response with impaired succinate signaling. On the other hand, while there was no difference at baseline, training-induced gains in grip strength were completely compromised in SUNCR1 KOs. In line with these observations, training resulted in increased neural-specific tubulin staining only in WT mice, while SUNCR1 KOs displayed downregulated fast MyHC isoforms as well as proteins involved in ECM organization relevant for force transmission. However, these results are in conflict with an earlier study, which found succinate supplementation to increase endurance exercise capacity and to induce an oxidative (*i.e.*, slow MyHC dominant) fiber type shift in a SUCNR1-dependent manner (Wang et al., 2019). Of note, while the effects of dietary succinate were attributed to myocellular SUCNR1 signaling, Reddy et al. (2020) were unable to detect SUCNR1 in cultured muscle cells or muscle fibers in tissue cross-sections, but found it to be highly expressed in resident stromal, endothelial and satellite cell populations, suggesting that succinate rather acts in a paracrine manner. The SUCNR1-dependent transcriptome in isolated cell populations immediately after an acute treadmill bout revealed decreased expression of transcripts involved in axon guidance, neuronal projections and muscle regeneration in SUCNR1 KO mice. Thus, despite the seemingly favored endurance adaptation, paracrine succinate signaling appears to be required for ECM remodeling and adaptions in muscle innervation in response to training. However, a thorough examination of changes in exercise performance, muscle function and oxidative capacity in SUCNR1 KOs is required to fully determine the role of succinate signaling in endurance training adaptation.

## MicroRNAs and Extracellular Vesicles

MicroRNAs (miRNAs) are small non-coding single-stranded RNA molecules that modulate gene expression at the *post*-transcriptional level. Extracellular miRNAs are either bound to protein complexes associated with high-density lipoproteins or located inside small extracellular vesicles (EVs). EVs are generated by most cell types and facilitate the exchange of miRNAs and other biological components among cells and tissues (Groot and Lee, 2020). Endurance exercise triggers a systemic EV release, and the amount as well as the specific composition of the miRNA cargo appears to be dependent on the exercise modality and intensity (Vechetti et al., 2021). Vechetti et al. (2021) further analyzed previously published miRNAs isolated from plasma EVs after a single bout of exercise and ran a prediction analysis in order to both determine possible targets of miRNA carrying EVs and to infer their potential systemic effects. The top two most significantly enriched biological processes revealed by gene ontology analysis were the response to reactive oxygen species and insulin secretion. This is in line with a recent study in mice showing that miRNA-containing EVs isolated from high-intensity interval-trained muscles improved glucose tolerance when administered to sedentary mice (Castaño et al., 2020). However, due to the inability to label and track EVs released from specific cells or tissues, the relative contribution of skeletal muscle to circulating EVs induced by exercise is still mostly speculative (Nederveen et al., 2020).

Besides the endocrine action of skeletal muscle-derived miRNAs as circulating EV cargo, *in vitro* evidence suggests that myofibers use miRNA secretion to regulate local processes such as myogenic differentiation and angiogenesis (Forterre et al., 2014; De Gasperi et al., 2017; Nie et al., 2019; Mytidou et al., 2021). Although it appears conceivable that a miRNA-based paracrine crosstalk could be involved in mediating muscular adaptations to endurance training (e.g., muscle fiber capillarization or ECM remodeling), the skeletal muscle miRNA field has only recently started to develop and hence there are currently very few studies investigating myofiber miRNA release in the context of endurance training adaptation.

## Factors Secreted by Resident or Infiltrating Cells in Skeletal Muscle

The complexity of skeletal muscle is often underappreciated and thus past efforts to identify factors mediating exercise-training adaptation have mainly focused on the secretome of myofibers. However, besides syncytial myofibers, skeletal muscle harbors a variety of mononucleated cell populations such as satellite cells (SCs), fibro-adipogenic progenitors (FAPs), macrophages, neutrophils, ECs, B-, T- and glial cells (Giordani et al., 2019). Exercise can induce changes in the proportion of these cell types as well as affect activation, polarization and secretory profiles (Rubenstein et al., 2020). Muscle-resident mononuclear cells contribute to the secretome of skeletal muscle and the adaptation to exercise training may depend on coordinated communication between the different cell types and muscle fibers, similar to developmental and regenerative processes. For example, IL-13 is secreted by type 2 innate lymphoid cells in endurance exercise, and induces signaling in myofibers that promotes an oxidative, high-endurance phenotype (Knudsen et al., 2020). Accordingly, besides reduced treadmill running capacity at baseline, the training-induced oxidative fiber type shift, improvements in mitochondrial respiration, endurance capacity and glucose tolerance were abolished in mice deficient for the *Il13* gene. Moreover, intramuscular delivery of IL-13 recapitulated the metabolic reprogramming induced by endurance training and increased exercise performance. Second, in recent years, histamine has emerged as a potentially important mediator of the exercise response, in both acute and chronic settings. In humans, histamine is released locally within exercised muscles and although not entirely clear, degranulating mast cells are a likely source of *post*-exercise histamine (Halliwill et al., 2013; Romero et al., 2017). Histamine-driven alterations in skeletal muscle gene expression could make up > 25% of the acute endurance exercise responsive transcriptome (Romero et al., 2016). Pharmacological inhibition of the histamine H_1_ and H_2_ receptors during 6 weeks of cycling interval training in healthy males resulted in impaired improvements of exercise capacity, glycemic control and vascular function (Van Der Stede et al., 2021). Finally, macrophage-derived growth differentiation factor 3 (GDF3) (Varga et al., 2016) and EC-secreted lactate (Zhang et al., 2020) help in muscle regeneration upon damage, thereby potentially contributing to training adaptation.

## Resistance Training and the Skeletal Muscle Secretome

Increasing or maintaining skeletal muscle mass and strength is not only essential for athletic performance, but also associated with increased quality of life, while conversely, a reduction in muscle mass and strength elevates the risk of (multi-)morbidity and all-cause mortality (Furrer and Handschin, 2019). Resistance training, which involves muscle contractions against external loads of a wide spectrum of ranges over several weeks or a lifetime, is an effective strategy to elicit substantial gains in, or to preserve skeletal muscle mass and strength in combination with adequate nutrition (Morton et al., 2018; Schoenfeld et al., 2021). Although the classically quantified adaptations to resistance training such as increased muscle volume, larger cross-sectional areas of pre-existing myofibers (hypertrophy) and increased muscle force appear trivial, the spectrum of structural and functional adaptations, especially those that underlie improvements in muscle force, is much more diverse. Neuronal and neuromuscular changes (e.g., motor output), connective tissue alterations and improved vascularization—to only name a few—are also elicited by resistance training, in both young and older individuals (Hughes et al., 2018). Although the resistance exercise stimuli, their molecular sensors as well as the downstream signaling events that lead to the above described phenotypic alterations induced by resistance training are still poorly characterized (Wackerhage et al., 2019), factors secreted by skeletal muscle fibers could play a key role in modulating neuromuscular plasticity in response to resistance training. Therefore, in the following sections, we provide an overview on resistance exercise-induced factors of the skeletal muscle secretome with potential functions in mediating muscular and systemic adaptations to resistance training through autocrine, paracrine or endocrine actions.

### IGF-1

IGF-1 is an extensively studied regulator of muscle growth, differentiation and regeneration. Upon binding to its receptor, IGF-1 induces phosphatidylinositol 3-kinase (PI3K)/Akt-dependent pathways, which subsequently lead to the activation of mTORC1 and p70S6 kinase, key regulators of protein synthesis and muscle growth, at least in certain contexts and paradigms (Goodman, 2019). Endocrine-acting IGF-1 is primarily secreted by the liver upon growth hormone (GH) stimulation, while skeletal muscle expresses the two mainly auto- and paracrine-acting isoforms IGF-1Ea and mechano-growth factor (MGF, or IGF-1Eb in rodents and IGF-1Ec in humans), of which MGF is most responsive to mechanical signals (Goldspink, 2005; Barton, 2006). PGC-1α4, a shorter PGC-1α isoform, which induces a hypertrophic phenotype when overexpressed in skeletal muscle of mice, has been proposed to act in part through IGF-1 induction and myostatin suppression (Ruas et al., 2012). Systemic IGF-1 administration is sufficient to induce muscle hypertrophy and resistance training-induced gains can be further potentiated when combined with IGF-1 treatment (Lee et al., 2004). However, a functional IGF-1 receptor appears to be dispensable for muscle hypertrophy to occur in a muscle-overload context (Spangenburg et al., 2008). Moreover, although acute resistance exercise increases systemic levels of IGF-1 and other anabolic hormones in human participants, acute raises in circulating IGF-1 after resistance exercise bouts neither enhance muscle protein synthesis nor correlate with skeletal muscle hypertrophy and strength gains following training (West et al., 2009; Morton et al., 2016). The relative contribution of skeletal muscle-derived IGF-1 to the systemic increase in response to resistance exercise, however, has yet to be assessed. Muscle IGF-1 mRNA and protein levels increase in response to both long-term resistance training in humans and in synergist ablation-induced muscle overload in rats (Adams and Haddad, 1996; Hanssen et al., 2013). Thus, even if the systemic fluctuations of IGF-1 induced by resistance exercise are negligible for the training outcome, muscle-derived IGF-1 may still have local and compared to non-muscle IGF-1, perhaps diverging effects. For example, muscle IGF-1 may regulate muscle fiber hypertrophy or repair in a paracrine manner by stimulating SC proliferation and incorporation into muscle fibers. Accordingly, increased DNA content was observed in muscles injected with IGF-1 (Adams and Mccue, 1998) and IGF-1 co-localization with Pax7, a marker for activated SCs, was enhanced upon an acute bout of resistance exercise (Grubb et al., 2014). However, whether SC activating IGF-1 is myofiber-derived in this context is difficult to estimate, as other cell types are also likely to be involved. Besides SCs themselves as a potential source, IGF-1 derived from monocytes/macrophages is known to be crucial during ECM remodeling and regeneration from muscle injury (Tonkin et al., 2015). Another layer of complexity in the regulation of IGF-1 signaling in response to exercise is added by the array of IGF-1 binding proteins that can either inhibit or promote IGF-1 bioavailability and physiological activity (Allard and Duan, 2018).

Finally, as IGF-1 is positively associated with bone mineralization (Breen et al., 2011), IGF-1 could also be involved in muscle-bone crosstalk by acting on IGF-1 receptors in the periosteum (Rittweger, 2008; Hamrick et al., 2010). However, even though skeletal muscle-specific overexpression of IGF-1 results in bigger bones (Banu et al., 2003), and a potential endocrine action of exercise-induced muscular IGF-1 release is at least conceivable, a direct link between resistance training and bone-specific adaptations has yet to be investigated.

### VEGF

Following both acute resistance exercise and hypertrophy-inducing resistance training, VEGF mRNA and protein levels increase in skeletal muscle, indicating that VEGF plays a role in the growing muscle (Richardson et al., 2000; Gavin et al., 2007; Kon et al., 2014). In cultured muscle cells, VEGF treatment induced myotube hypertrophy, whereas VEGF inhibitors reduced myotube size (Bryan et al., 2008). Moreover, in mice, muscle-specific VEGF gene ablation blunts both muscle hypertrophy and strength gains in a functional overload context (Huey et al., 2016). Interestingly, the VEGF transcriptional response after an acute bout of resistance exercise seems to be attenuated in resistance-trained compared to untrained muscles (Richardson et al., 2000), which may indicate that capillarization is substantially stimulated at an early stage of training to allow subsequent muscle fiber growth, while the signal flattens out when the hypertrophy rate is slowing down. However, VEGF induction may also depend on the exercise protocol as others found non-significant or a similar increase in acute induction of VEGF pre- and *post*-resistance training (Coffey et al., 2006; Trenerry et al., 2011). It is currently not well understood how skeletal muscle synthesizes and secretes VEGF in response to hypertrophic stimuli, but interestingly, *in vitro* studies found enhanced VEGF synthesis and secretion by C2C12 myotubes upon IGF-1 treatment and Akt activation (Takahashi et al., 2002; Florin et al., 2020). Similarly, injection of constitutively active Akt into the *M. gastrocnemius* of mice enhanced both circulating and local VEGF levels (Takahashi et al., 2002). Future studies will have to test whether IGF-1/Akt-mediated VEGF induction plays a role in the physiological adaptation to resistance training or whether the stimuli and pathways leading to VEGF secretion and/or muscle fiber capillarization in general overlap with those of endurance exercise.

### Myostatin

Myostatin is a highly conserved member of the TGF-β protein family and is mainly expressed in skeletal muscle (Thomas et al., 2000). The role of myostatin as a negative regulator of muscle growth was first discovered in 1997 by Mcpherron et al. (1997), who observed an approximately twofold increase in muscle mass in myostatin KO mice. Likewise, antagonism of muscle myostatin signaling in mice substantially increases muscle mass, further highlighting the growth-limiting effects of myostatin (Lee and Mcpherron, 2001; Amthor et al., 2004). Intriguingly, besides its occurrence in cattle, sheep and dogs, a loss-of-function mutation in the myostatin gene may also occur spontaneously in humans, as it happened in a young boy displaying a pronounced hypertrophic phenotype (Schuelke et al., 2004).

Upon secretion, myostatin binds to the activin receptor type IIB (ActRIIB) to build a complex with activin receptor-like kinase 4 or 5 (ALK4/5) on muscle fiber membranes and SCs, which in turn activates small worm phenotype/mothers against decapentaplegic (SMAD) transcription factors that both suppress PI3K/Akt-mediated mTORC1 signaling and induce FoxO-dependent protein degradation pathways (Han et al., 2013). However, how mTORC1 is regulated by myostatin has yet to be determined. The muscle mass increase in myostatin KO animals results from both hyperplasia and hypertrophy of muscle fibers. Hyperplasia is primarily driven during development *in utero*, while hypertrophy is thought to be the predominant process responsible for myostatin-related postnatal muscle growth, at least in rodents and in humans. Initial studies on how myostatin negatively regulates muscle mass pointed towards SC regulation, as myostatin KO mice have an increased rate of SC proliferation and incorporation into pre-existing myofibers (Mccroskery et al., 2003; Wagner et al., 2005). However, more recent studies found that SC activation upon myostatin inhibition does not precede myofiber hypertrophy and thus concluded that myostatin regulates hypertrophy also in a SC-independent manner (Lee et al., 2012; Wang and Mcpherron, 2012). Of note, the larger muscle mass in myostatin KOs does not translate into increased strength (Amthor et al., 2007), which might in part explain why pharmacological inhibition of myostatin signaling largely failed to mitigate functional impairments in muscle wasting diseases and sarcopenia (Rooks et al., 2020).

Antagonistic to growth hormones, myostatin contributes to the determination of a set point of pre-programmed, yet adaptable level of muscle mass. Inconclusive results have been reported regarding the regulation of myostatin expression following chronic resistance exercise and the increase in muscle mass, and at least in some studies, myostatin expression even increases after acute exercise bouts (Domin et al., 2021). It is therefore unclear whether a potential transient or chronic suppression of *post*-exercise and *post*-training levels, respectively, is part of the training effect. Signals affecting the myostatin pathway could also be involved in resistance training adaptation. For example, resistance exercise-induced cleavage of intracellular notch domains overrides the inhibitory effect of myostatin by inhibiting SMAD proteins (Mackenzie et al., 2013). Finally, besides its role in keeping muscle mass in check, myostatin also suppresses bone development, as evidenced by increased bone formation in myostatin-deficient mice (Hamrick, 2003; Dankbar et al., 2015). In the bone, the analogous ActRIIB/SMAD pathway is involved in osteoclastogenesis and binding of myostatin induces osteoclast formation and thus bone erosion (Bialek et al., 2014; Dankbar et al., 2015). Resistance training is a strong stimulus to boost bone mineral density and counteract osteoporosis, potentially at least in part mediated by reduced myostatin signaling (Zamoscinska et al., 2020), even though an ever expanding complex system of multidirectional signaling between muscle (myokines), bones (osteokines), liver (hepatokines) and adipose tissue (adipokines) certainly contributes to this and other organ crosstalk in exercise (Gonzalez-Gil and Elizondo-Montemayor, 2020).

### Follistatin

Follistatin is a secreted glycoprotein expressed in various cell types, including skeletal muscle (Görgens et al., 2013). Endogenous extracellular follistatin binds myostatin, thereby preventing myostatin-ActRIIB/ALK4/5 receptor interaction, and consequently inhibiting the anti-anabolic effects of myostatin (Lee and Mcpherron, 2001). While myostatin loss (Mcpherron et al., 1997) and muscle-specific follistatin overexpression (Lee and Mcpherron, 2001) induce significant muscle hypertrophy on their own, their phenotypes appear to be additive. Myostatin loss alone increases muscle mass by about twofold, but intriguingly, combined myostatin KO and follistatin overexpression results in quadrupling of muscle mass (Lee, 2007), indicating that follistatin also acts independent of myostatin. In fact, follistatin-induced inhibition of SMAD3 activity, which leads to induction of protein synthesis in skeletal muscles *via* Akt/mTORC1/S6K signaling, occurs also through inhibition of activin, another follistatin- and ActRIIB-ligand implicated in both muscle mass and strength loss in various conditions (Gilson et al., 2009; Winbanks et al., 2012). Although there are not many human training studies available, both serum follistatin as well as skeletal muscle follistatin mRNA levels seem to be upregulated upon long-term resistance training (Laurentino et al., 2012; Negaresh et al., 2019). The observation that follistatin is not only elevated in muscle, but also in the circulation, leads to the question whether muscle-derived follistatin—besides controlling skeletal muscle adaptation in a local manner—also operates systemically. However, the bulk of circulating follistatin is not muscle- but liver-derived, indicating that endocrine follistatin is rather a hepatokine (Hansen et al., 2016). In this regard, a potential function of circulating follistatin in glycemic control by modulating insulin action in skeletal muscle has recently been proposed (Han et al., 2019).

### Decorin

Decorin is an extracellular matrix proteoglycan involved in the skeletal muscle hypertrophic response. Although *in vitro* evidence suggests that decorin is secreted by myotubes and might be involved in protein synthesis pathways, partly by inhibiting myostatin actions similar to follistatin, the influence of a long-term resistance training program on muscle decorin levels has not been studied so far (Guiraud et al., 2012; Sun et al., 2013; Kanzleiter et al., 2014).

### Matrix Metalloproteinase-2

Matrix metalloproteinases (MMPs) are a family of enzymes involved in ECM remodeling (Nagase and Woessner, 1999). *In vitro* studies indicate that around 30% of skeletal muscle MMP-2 are actively secreted into the ECM and that MMP-2 has an important function in muscular differentiation, regeneration and repair (Chen and Li, 2009; Ren et al., 2019). Anomalous ECM remodeling, such as connective tissue accumulation, affects skeletal muscle function and is often observed in aging (Wood et al., 2014), while aberrant accumulation of connective tissue can be effectively prevented with resistance training (Guzzoni et al., 2018). Moreover, muscle ECM remodeling is an integral part of the resistance training response that follows a biphasic course in which initial catabolic processes, guided by a transient glycoprotein matrix, are followed by anabolic processes to reinforce the intramuscular connective tissue structure (Csapo et al., 2020). Evidence further indicates that long-term resistance training significantly elevates muscle MMP-2 levels and thus, a potential link between MMP-2 and resistance training-induced ECM remodeling of skeletal muscle has been suggested (Deus et al., 2012; Shiguemoto et al., 2012; Souza et al., 2014; De Sousa Neto et al., 2018; Guzzoni et al., 2018). Significantly reduced ECM remodeling concomitant with impaired muscle hypertrophy and force production in MMP-2 KO mice in response to synergist ablation-induced functional overload further supports the importance of MMP-2 action during resistance training adaptation (Zhang et al., 2014). Interestingly, Akt/mTORC1 signaling is unaffected in MMP2-KO mice, indicating that disruption of the ECM remodeling alone is sufficient to reduce functional overload-induced muscle hypertrophy. In addition, skeletal muscle-secreted MMP-2 is involved in exercise-induced skeletal muscle angiogenesis and potentially muscle-bone crosstalk, although it is not entirely clear whether these effects are indeed mediated by muscle-derived MMP-2 (Haas et al., 2000; Hamrick, 2012; Chen et al., 2021).

### Secreted Protein Acidic and Rich in Cysteine

Secreted protein acidic and rich in cysteine (SPARC) is a secretory matricellular protein involved in mediating cell-ECM interactions (Brekken and Sage, 2000). SPARC expression has been described in many tissues undergoing repair or remodeling and accordingly, both SPARC mRNA and protein expression were elevated in human muscle biopsies after long-term resistance training (Brekken and Sage, 2000; Norheim et al., 2011). Interestingly, despite being present in both myofibers and capillary ECs, training-induced SPARC proteins strongly accumulate adjacent to the plasma membrane of myofibers, indicating increased communication between muscle cells and the ECM in response to resistance training (Norheim et al., 2011). Although the hypertrophy-promoting signals of SPARC are not well understood, muscle atrophy, resulting from muscle specific loss of SPARC, could be due to enhanced TGF-β signaling (Nakamura et al., 2013). Besides its anti-atrophic effects, studies in lens ECs provide evidence for an additional hypertrophy-promoting mechanism of SPARC. In this *in vitro* system, SPARC acts on the transmembrane receptor integrin β1, which in skeletal muscle plays a critical role in myofiber differentiation, skeletal muscle innervation and sensing of mechanical stimuli (Schwander et al., 2004; Weaver et al., 2008; Boppart and Mahmassani, 2019). Thus, it can be speculated that SPARC induces skeletal muscle hypertrophy in part through autocrine actions *via* SPARC/integrin signaling. There are also other para- and/or endocrine signaling events that have been attributed to SPARC e.g., in bone homeostasis, adipose tissue turnover and tumorigenesis (Brekken and Sage, 2000; Nie and Sage, 2009). However, whether muscle-derived SPARC acts on these cell types in a para- and/or endocrine manner has yet to be investigated.

### IL-6

Chronically elevated levels of circulating IL-6 or chronic IL-6 administration directly into skeletal muscle lead to substantial muscle atrophy (Tsujinaka et al., 1996; Haddad et al., 2005). However, IL-6 expression is increased during recovery from cast-immobilization induced atrophy in rats (Childs et al., 2003), and IL-6-deficient mice display prolonged recovery from atrophy induced by hind limb suspension (Washington et al., 2011). In the latter study, IL-6 KOs also showed a blunted transcriptional induction of the myogenesis-regulating transcription factors MyoD and myogenin as well as impaired IGF-1 transcription and Akt/mTORC1 signaling after 1 day of recovery. By comparing WT and IL-6 KO mice combined with *in vitro* studies, IL-6 was shown to control SC proliferation and myonuclear accretion in functional overload-induced skeletal muscle hypertrophy, most likely by activating the IL-6 downstream target STAT3 (Serrano et al., 2008). However, using the same approach, White et al. (2009) observed similar gains in myofiber cross-sectional area in WT and IL-6-deficient mice, while the increase in muscle wet mass was even larger in IL-6 KO animals. By means of histological analyses, the extra gains in muscle mass could be attributed to a larger increase in non-contractile tissue. Accordingly, mRNA levels of procollagen-1, IGF-1, and TGF-β were highly induced in IL-6-deficient mice, whereas the elevation of MyoD, required for myogenic differentiation (Yu et al., 2021), was attenuated compared to WT animals. Collectively, these studies indicate that IL-6 is important for SC-mediated hypertrophy and may be involved in intramuscular connective tissue remodeling e.g., to prevent fibrosis in response to increased mechanical loading.

In the context of resistance exercise, circulating and skeletal muscle IL-6 concentrations are highly induced by an acute bout of resistance exercise in recreationally active individuals (Vella et al., 2012; Benini et al., 2015). Moreover, acute resistance exercise increases STAT3 activity in skeletal muscle (Trenerry et al., 2007), a response that seems to be preserved after training (Trenerry et al., 2011). In ladder climbing rats and resistance exercising young men, resting IL-6 protein levels are elevated in skeletal muscle following 10-12 weeks of training (Trenerry et al., 2011; Begue et al., 2013; Jung et al., 2015). The systemic increase following acute bouts as well as resting circulating IL-6 levels, on the other hand, appear to be reduced after long-term resistance training (Donges et al., 2010; Libardi et al., 2012; Ho et al., 2013; Azizbeigi et al., 2015; Forti et al., 2017). Although the transient increase in local IL-6 concentrations following acute resistance exercise-induced myotrauma is associated with STAT3 and SC activation (Mckay et al., 2009; Toth et al., 2011), the contribution of IL-6 to regenerative processes, SC-dependent hypertrophy, or both, in resistance training adaptation is still incompletely understood. Moreover, whether IL-6 activates the JAK/STAT3 signaling pathway exclusively in SC or also in muscle fibers in an autocrine manner remains to be determined.

### Leukemia Inhibitory Factor

Leukemia inhibitory factor (LIF) is a member of the IL-6 cytokine superfamily, a group of structurally and functionally related proteins (also known as neuropoietins or pg130 cytokines), and is expressed in various tissues including skeletal muscle (Heinrich et al., 1998; Metcalf, 2003). In human myotubes, production and secretion of LIF is enhanced upon electrical stimulation, potentially involving PI3K and Akt/mTORC1 signaling (Broholm et al., 2011). In rodents, LIF protein levels were upregulated in the *M. plantaris* by functional overload-induced hypertrophy (Sakuma et al., 2000). Moreover, in LIF KO animals, the hypertrophic response is missing, but can be rescued with systemic application of LIF, indicating that LIF is required for hypertrophy in an overload context (Spangenburg and Booth, 2006). Interestingly, LIF-dependent muscle growth may be fiber type-specific, as hypertrophy of the slow MyHC-dominant *M. soleus* is only delayed, but not completely abolished in LIF KO mice. Systemic LIF administration for 4 weeks increases *M. soleus* mass in rats, whereas hypertrophy in the *M. extensor digitorum longus*, a prototypical fast MyHC-muscle, required simultaneous supplementation with clenbuterol (a β2-adrenoreceptor agonist) to respond in a significant manner, further supporting the muscle fiber-type-specific actions of LIF (Gregorevic et al., 2002). In humans, muscle LIF mRNA is upregulated upon a single bout of resistance exercise, while circulating LIF levels remain unchanged (Broholm et al., 2011).

Similar to IL-6, LIF promotes hypertrophy in a paracrine manner by stimulating SC proliferation through the JAK/STAT3 signaling pathway, as indicated by *in vitro* studies (Spangenburg and Booth, 2002). In addition, induction of the transcription factors JunB and c-Myc may underlie the enhanced myoblast proliferation triggered by LIF as observed in cultured human myotubes (Broholm et al., 2011). However, emerging evidence suggests that LIF regulates skeletal muscle plasticity in a more complex manner. For example, LIF is essential in the maintenance of the NMJ, both pre- and *post*-synaptically, which raises the possibility that LIF regulates NMJ/MN remodeling during resistance training adaptation (Hunt and White, 2016). Furthermore, a designer cytokine with the ability to signal *via* LIF receptor/gp130 in an IL-6 receptor-dependent manner was tested for the treatment of T2D (Findeisen et al., 2019). Intriguingly, besides the positive effects on all major hallmarks of the metabolic syndrome in obese mice, the treatment also prevented the loss of, or even increased skeletal muscle mass at least in part by activating the transcriptional coactivator and Hippo pathway effector yes-associated protein 1 (YAP1) in skeletal muscle. These findings provide an exciting additional and notably SC-independent mechanism for how IL-6 receptor/gp130 and/or LIF receptor signaling could potentially mediate muscle hypertrophy or mitigate muscle loss in various conditions. Clinical trials will have to reveal the translational potential of these observations as well as determine the LIF-dependent skeletal muscle adaptations to resistance training.

### IL-8

Besides its proposed role in endurance exercise, IL-8 may also be involved in mediating muscular adaptations to resistance training. Both skeletal muscle IL-8 mRNA and protein levels are enhanced after an acute bout of resistance exercise in human participants while circulating levels remain unchanged (Buford et al., 2009; Della Gatta et al., 2014a). Moreover, muscle IL-8 mRNA and peptide levels are significantly increased following a long-term resistance-training regimen (Nieman et al., 2004; Della Gatta et al., 2014b). Interestingly, in contrast to a single bout of resistance exercise, resistance training also upregulates circulating IL-8 levels in some studies (Forti et al., 2016, 2017). However, it remains unsolved whether skeletal muscle fibers are responsible for the long-term increase in plasma IL-8, or whether this is due to IL-8 production in other cell types in skeletal muscle or even in other sites in the body.

Even though the function of muscle-derived IL-8 in the context of resistance training adaptation is not exactly known, it is thought that IL-8—similar to its role in endurance adaptation—promotes angiogenesis in loaded muscles. Interestingly however, recent *in vitro* evidence suggests that IL-8 might further regulate muscle miRNAs, particularly miR-338 and miR-376, which are involved in regulating skeletal muscle growth in neonatal piglets (Mcdaneld et al., 2009; Milewska et al., 2019). In addition, when present in the extracellular environment of rat myoblasts, IL-8 induces myogenic transcription, Akt phosphorylation and FoxO3 degradation, thereby inhibiting muscle cell proteolysis (Milewska et al., 2019). However, more research, in particular *in vivo* studies, is necessary to confirm the potential anti-catabolic effects of IL-8 and to further substantiate the suggested role in angiogenesis.

### Other Interleukins

Besides IL-6 and IL-8, other interleukins have been proposed to be involved in mediating resistance-training adaptation. IL-7 is a muscle cell-secreted cytokine with potential anabolic functions, as muscle IL-7 mRNA was upregulated in human *M. vastus lateralis* and *M. trapezius* after a resistance-training program in young men (Haugen et al., 2010). However, due to the lack of follow-up studies, the role of IL-7 peptides in mediating hypertrophy or other types of resistance-training adaptations remains elusive.

Based on the observation that IL-15 is highly expressed in skeletal muscle (Grabstein et al., 1994) and experiments in cultured muscle cell lines (Quinn et al., 2002), IL-15 was initially believed to act as an anabolic factor for skeletal muscle. Subsequent studies with either IL-15 administration or overexpression however, have largely failed to increase muscle mass in healthy rodents. On the contrary, these interventions rather seem to promote an oxidative phenotype of skeletal muscle (Pistilli and Quinn, 2013). Although these experiments may not mimic the physiological dynamics of IL-15 *in vivo*, data from resistance exercising healthy humans so far could not provide strong evidence to support the notion of IL-15 having anabolic properties (Nielsen et al., 2007; Pérez-López et al., 2018). Thus, more research is required to determine the physiological role of IL-15 in skeletal muscle, with particular focus on its cognate soluble and membrane-bound receptors (Nadeau and Aguer, 2019).

IL-4 and IL-13 were found to regulate myoblast fusion in cultured muscle cells (Horsley et al., 2003; Jacquemin et al., 2007). In young men, 6 weeks of resistance training increased gene expression of IL-4 and IL-13 as well as both their receptors in arm extensor muscles (Prokopchuk et al., 2007). In contrast, resting peptide levels of muscle IL-4 and IL-13 are unchanged following resistance training, while IL-4 appears to be induced 2 h after an acute bout of resistance exercise, following 12 weeks of resistance training (Prokopchuk et al., 2007; Della Gatta et al., 2014b; Jung et al., 2015).

In summary, the literature on the interleukins described above in resistance-training adaptation is still rudimentary and therefore, further studies are necessary to draw firm conclusions on their potential functions in this context. Moreover, the actual source of these cytokines upon resistance exercise has to be determined, in particular for IL-4 and IL-13, as they likely originate also from either resident or infiltrating mononucleated cell types.

## miRNAs

Similar to endurance exercise, resistance exercise increases circulating miRNAs in a protocol-dependent manner (Cui et al., 2017). Myogenic progenitor cells regulate the muscle ECM by secreting EVs containing miR-206, which represses ribosomal binding protein 1 (a master regulator of collagen biosynthesis) in interstitial fibrogenic cells to prevent excessive ECM deposition in response to synergist ablation-induced overload of the *M. plantaris* (Fry et al., 2017). The miR-199-3p enhances myogenic differentiation and muscle regeneration, and, when administrated to aged mice, induced muscle fiber hypertrophy and delayed loss of muscle strength (Fukuoka et al., 2021). Moreover, mdx mice, a model of Duchenne muscular dystrophy, improved muscle strength upon treatment with miR-199 mimics. As the muscle fiber-derived miRNA field is still emerging, the future will likely reveal the potential regulatory capacity of secreted miRNAs in resistance training adaptation.

## Concluding Remarks

Exercise training adaptation is the result of the repeated application of exercise stimuli that elicit multi-organ responses. During this process, skeletal muscle, the heart and other organs integrate signals arising from both cell-intrinsic perturbations and external cues. While a basic understanding of many mechanisms that are important in acute exercise bouts exists, our mechanistic knowledge about chronic training adaptation is rudimentary, both for endurance as well as resistance exercise. Along the same lines, most myokines have been identified and studied in the context of acute, and mostly endurance exercise bouts. It thus is not surprising that the study of auto-, para- and endocrine signaling of myokines in training is still in its infancy. In this review, we have summarized the current data on myokines and other muscle-derived factors that could potentially be linked to training adaptation, trying to not only include, but also contrast the findings in endurance and resistance exercise paradigms. Notably, for almost all of those, data are scarce, and it often is not clear whether the relative and absolute levels, release and elimination kinetics, or other parameters are indeed changed in response to training in humans. Alternatively, it is conceivable that the repeated release of secreted factors and their downstream signaling over time contribute to training adaptation in a cumulative manner. Studies in human volunteers is hampered by the difficulty to track the source of secreted factors, and thus attribution to myokine action remains elusive since most of these factors are also produced by other cell types and tissues. Moreover, circulating concentrations often are very low, and therefore, conventional detection methods, might not be sufficiently sensitive, produce unspecific and unreliable results, or might not even exist. Mouse models are more suited for causative studies of specific factors. However, caution is advised in the use of pre-/perinatal or juvenile gain- and loss-of-function models. Inducible mouse models might be compromised by the effect of tamoxifen or doxycycline on skeletal muscle and the chance of incomplete transgenesis. Finally, gain-of-function models should overexpress the gene of interest within a physiological range and ideally in a transient manner as observed in exercise, to avoid artificial effects of superphysiological and constitutively elevated levels. Thus, optimally, the study of the skeletal muscle secretome in acute exercise, training, as well as pathological settings should be done in different models and paradigms, and carefully validated on the mechanistic level. Besides these challenges in discovery and validation, the potential therapeutic exploitation of myokines faces several obstacles (Whitham and Febbraio, 2016). Among those, issues with stability, immunogenicity and toxicity as well as achieving cell- and/or tissue-specific targeting to avoid side effects are key limitations. If successfully overcome, however, myokines have an enormous potential as agents to improve muscle function in healthy individuals as well as in many different diseases (Pedersen and Saltin, 2015; Pedersen, 2019).

## Author Contributions

All authors drafted the manuscript, prepared the figure, edited and approved the final version of the manuscript.

## Conflict of Interest

The authors declare that the research was conducted in the absence of any commercial or financial relationships that could be construed as a potential conflict of interest.

## Publisher’s Note

All claims expressed in this article are solely those of the authors and do not necessarily represent those of their affiliated organizations, or those of the publisher, the editors and the reviewers. Any product that may be evaluated in this article, or claim that may be made by its manufacturer, is not guaranteed or endorsed by the publisher.
